# Airway management in a Helicopter Emergency Medical Service (HEMS): a retrospective observational study of 365 out-of-hospital intubations

**DOI:** 10.1186/s12873-022-00579-8

**Published:** 2022-02-08

**Authors:** Urs Pietsch, Raphael Müllner, Lorenz Theiler, Volker Wenzel, Lorenz Meuli, Jürgen Knapp, Stephen J. M. Sollid, Roland Albrecht

**Affiliations:** 1 Department of Anaesthesiology and Intensive Care Medicine, Cantonal Hospital St, Gallen, St. Gallen, Switzerland; 2 Swiss Air-Ambulance, Rega (Rettungsflugwacht/Guarde Aérienne), Zürich, Switzerland; 3 Department of Emergency Medicine, Inselspital, Bern University Hospital, University of Bern, Freiburgstrasse, 3010 Bern, Switzerland; 4 Department of Anaesthesiology, Cantonal Hospital Luzern, Luzern, Switzerland; 5 Department of Anaesthesiology, Kantonsspital Aarau, Aarau, Switzerland; 6 Department of Anaesthesiology and Intensive Care Medicine, Friedrichshafen Regional Hospital, Friedrichshafen, Germany; 7 Department of Vascular Surgery, University Hospital Zürich, Zürich, Switzerland; 8 Department of Anaesthesiology and Pain Medicine, Inselspital, Bern University Hospital, University of Bern, Bern, Switzerland; 9 Norwegian Air Ambulance Foundation, PB 414 Sentrum, 0103 Oslo, Norway; 10 Faculty of Health Sciences, University of Stavanger, PB 8600, 4036 Stavanger, Norway

**Keywords:** HEMS, Airway management, Difficult intubation, Out-of-hospital intubation, Difficult airway management

## Abstract

**Background:**

Airway management is a key skill in any helicopter emergency medical service (HEMS). Intubation is successful less often than in the hospital, and alternative forms of airway management are more often needed.

**Methods:**

Retrospective observational cohort study in an anaesthesiologist-staffed HEMS in Switzerland. Patient charts were analysed for all calls to the scene (*n* = 9,035) taking place between June 2016 and May 2017 (12 months). The primary outcome parameter was intubation success rate. Secondary parameters included the number of alternative techniques that eventually secured the airway, and comparison of patients with and without difficulties in airway management.

**Results:**

A total of 365 patients receiving invasive ventilatory support were identified. Difficulties in airway management occurred in 26 patients (7.1%). Severe traumatic brain injury was the most common indication for out-of-hospital Intubation (*n* = 130, 36%). Airway management was performed by 129 different Rega physicians and 47 different Rega paramedics. Paramedics were involved in out-of-hospital airway manoeuvres significantly more often than physicians: median 7 (IQR 4 to 9) versus 2 (IQR 1 to 4), *p* < 0.001.

**Conclusion:**

Despite high overall success rates for endotracheal intubation in the physician-staffed service, individual physicians get only limited real-life experience with advanced airway management in the field. This highlights the importance of solid basic competence in a discipline such as anaesthesiology.

## Background

Where available, helicopter emergency medical services (HEMS) provide fast and effective treatment and transport of severely ill or injured patients, even in areas with difficult access such as alpine regions. Among the critical life-saving skills provided by HEMS, advanced airway management has long been considered a core skill [1]. However, out-of-hospital endotracheal intubation (ETI) success rates vary between HEMS services and systems, depending on levels of competence and experience [2, 3]. In the hands of well-trained and experienced personnel, rates of successful intubation on the scene are comparable to rates in the hospital [4, 5].

Factors that seem to facilitate high success rates are ETI provided by physicians, and trans-oral, rapid-sequence and drug-facilitated ETI [3, 4]. Success rates are lower in certain patient subgroups, especially patients suffering trauma and cardiac arrest [4]. Complication rates are also generally higher and first-pass success (FPS) rates lower in the out-of-hospital setting, which may have an impact on patient outcome in the long run [4–6].

Typically, exclusive factors for the out-of-hospital setting play a role, such as early management of traumatic facial injuries and of environmental factors like weather, light and access to the patient [7]. Failed initial intubation attempts usually lead to optimisation of patient positioning, use of intubation assist devices, or use of alternative airway management devices [8]. The use of devices such as video laryngoscopes (VL) may further facilitate success rates and reduce complication rates [9]. Advanced prehospital airway management therefore still needs close oversight, and HEMS systems should be aware of their success rates and complication rates.

The goal of this study was to critically review cases of advanced airway management encountered by Rega HEMS, identify the success rates and difficulties recorded, and describe the techniques that eventually secured the airway.

## Methods

### Setting, definitions and ethics

In Switzerland, five organizations provide 24/7 physician-staffed HEMS operations. Two thirds of these operations are pre-hospital retrievals (primary missions) and one third are inter-hospital transfers (secondary missions). Rega operates 12 helicopter bases, located throughout Switzerland, and helicopter teams can reach any location in the operational area within 15 min of flight time, day or night, provided the weather conditions are met [10].

The HEMS crew typically includes a pilot, a HEMS physician, and a paramedic who serves as technical crew member, hoist operator and as assistant in the airway management process. HEMS physicians are required to be board certified in Anaesthesiology. Several HEMS physicians hold an additional certification in intensive and critical care medicine and/or mountain emergency medicine. These physicians are experienced in emergency airway management, including difficult airway management and paediatric anaesthesia (paediatric advanced life support providers). The Rega standard of advanced airway management is ETI performed as a rapid sequence induction and intubation without obligation to omit mask ventilation prior to ETI [11]. The equipment is standardised throughout the organisation, and includes special paediatric airway equipment.

In HEMS missions conducted by Rega, difficulties in airway management are defined as several reported attempts at intubation, the need for assist devices such as bougies, an intubating laryngeal mask airway, secondary placement of a supraglottic airway device, and cricotomy.

### Study population

For this retrospective observational cohort study, digital patient records of all consecutive primary HEMS missions conducted by Rega from June 1st, 2016, to May 1st, 2017, were screened. The protocols of all patients with reported use of any type of ventilatory support were analysed by hand. Patients with invasive ventilatory support such as tracheal intubation, supraglottic devices, and front-of-neck access were included. Patients with non-invasive ventilatory support (NIV) mask ventilation without attempted invasive ventilatory support, and transfers between hospitals were excluded from this analysis. In order to obtain a homogeneous sample regarding mission documentation, equipment used, and crew training, patients transported by Rega who underwent previous advanced airway management initiated by ground emergency medical services and terminated upon arrival of the HEMS team were also excluded.

### Outcomes

The primary outcome was first-pass success (FPS) and overall intubation success rate as defined by Frerk et al. [12]. Secondary outcomes included on-site mortality and mortality during transportation. Descriptive patient and mission characteristics included age, sex, the type of diagnosis (trauma, cardiovascular, neurologic, others), NACA score (National Advisory Committee for Aeronautics), the use and type of neuromuscular blocking agents (NMBA), cardiopulmonary resuscitation (CPR), the location where intubation was performed as a binary variable (i.e. remote terrain versus urban), and the time of the mission during the day as a binary variable (i.e. day between 6am and 8 pm). Further, the total number of intubations performed by an individual was recorded for both paramedics and physicians.

### Statistical analysis

Patients’ characteristics were summarised and presented in tables. Continuous variables were summarised by mean and standard deviation (SD) if normally distributed or by median and interquartile range (IQR) if skewed. Normality was visually inspected and formally tested using the Shapiro–Wilk test. Categorical variables were summarised with counts and percentages for each level of the variable. Continuous variables were compared using Student’s t-test if normally distributed or the Mann–Whitney U test if skewed. Categorical variables were compared using Pearson’s chi-squared test.

Exploratory analyses were performed to elaborate on the impact of factors that are potentially associated with a difficult airway, using a multivariate logistic regression model. The model included the a priori defined variables age, sex, *diagnosis*, *use of NMBA*, use of catecholamines, *CPR*, *NACA level, duration of the mission*, and *access to the patient*. All analyses were conducted with R-Studio, version 3.4.3, on MacOS version 10.15.7. p-values are two-sided with an α-level of 5%. All methods were performed in accordance with the relevant guidelines and regulations.

## Results

**Fig. 1:**
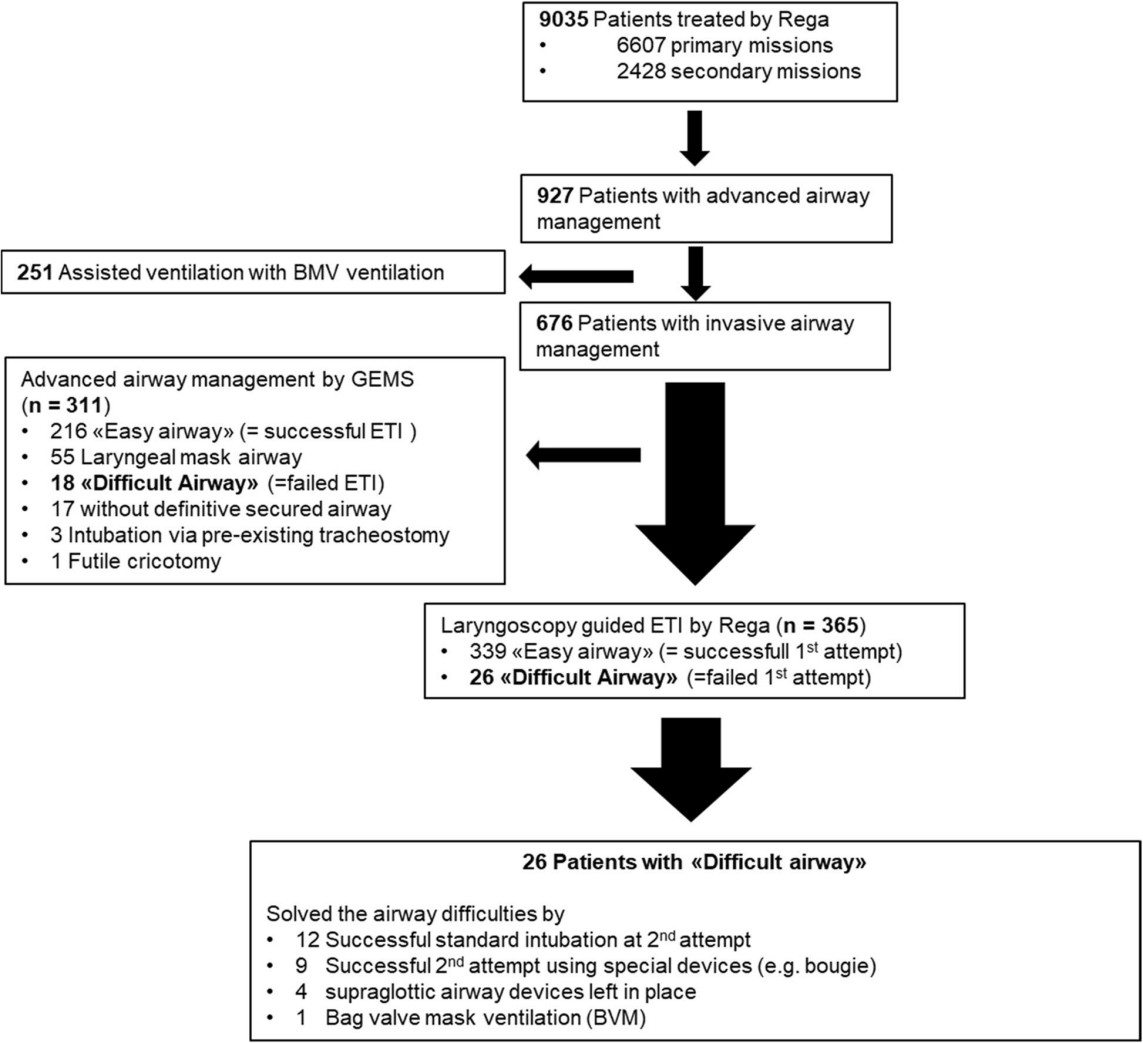
Patient flow chart

During the observation period from June 1st, 2016, until May 31st, 2017, Rega was deployed for a total of 9,035 missions. 2,428 secondary missions were excluded, and the remaining 6,607 primary missions were potentially eligible for analysis. In 927 missions, some type of airway management was identified. In 251 of these missions, it was short-term assisted ventilation using only bag-valve mask ventilation. In 311 missions, advanced airway management had already been initiated by ground emergency medical services upon arrival of the HEMS. A total of 365 patients received invasive airway management by HEMS crew, completing the study cohort (Fig. 1).

In 26 of the 365 patients (7.1%), difficulties in the airway management were reported. In the remaining 339 patients a first attempt at establishing an airway was successful, resulting in a 92.9% first-attempt success rate.

### Management of difficult airways

The Rega team solved the airway management difficulties using standard intubation (successful standard intubation on a 2^nd^ attempt) in twelve patients. In nine patients a 2^nd^ attempt intubation was successful using a bougie. Bag valve mask (BVM) ventilation was performed in one patient until arrival in the hospital after more than three failed intubation attempts. In four patients, the supraglottic airway device was left in place after a failed 2^nd^ intubation attempt (Fig. 1).

### Patient and mission characteristics

The mean age of the patients was 53.1 (SD: 22.5) years, and most of the patients were male (73.7%). Airway management was uneventful in all 39 paediatric patients. Six children were aged 0–1 years, 18 were aged 2–6 years, and 15 were aged 7–15 years. There were no significant differences in these baseline characteristics between patients with difficult airways and patients with successful first-attempt airway management (see Table 1).

**Table 1:** Baseline characteristics

	**Easy Airway** ***N* = 339**	**Difficult Airway** ***N* = 26**	**Total** ***N* = 365**	*p* value
**Age in years**				0.756
Mean (SD)	53.2 (22.5)	51.7 (22.1)	53.1 (22.5)	
Range	0.0—96.0	4.0—83.0	0.0—96.0	
**Female Sex**	88 (26.0%)	8 (30.8%)	96 (26.3%)	0.591
**Indication / Diagnosis**				0.190
**Trauma**	140 (41.3%)	16 (61.5%)	156 (42.7%)	
Traumatic brain injury	117 (83.6%)	13 (81.2%)	130 (83.3%)	
Chest	7 (5%)	2 (12.5%)	9 (5.8%)	
Spinal injury	3 (2.1%)	0	3 (1.9%)	
Other trauma	13 (9.3%)	1 (6.3%)	14 (9%)	
**Cardiovascular**	111 (32.7%)	4 (15.4%)	115 (31.5%)	
Heart failure	98 (88.3%)	4 (100%)	102 (88.7%)	
Respiratory failure	8 (7.2%)	0	8 (6.9%)	
Other cardiovascular	5 (4.5%)	0	5 (4.4%)	
**Neurological**	54 (15.9%)	4 (15.4%)	58 (15.9%)	
Stroke	36 (66.7%)	3 (75%)	39 (67.2%)	
Status epilepticus	10 (18.5%)	1 (25%)	11 (19%)	
Other neurological	8 (14.8%)	0	8 (13.8%)	
**Other**	34 (10.0%)	2 (7.7%)	36 (9.9%)	
**CPR**	96 (28.3%)	6 (23.1%)	102 (27.9%)	0.566
**NACA**				0.320
4	17 (5.0%)	0 (0.0%)	17 (4.7%)	
5	177 (52.2%)	18 (69.2%)	195 (53.4%)	
6	80 (23.6%)	4 (15.4%)	84 (23.0%)	
7	65 (19.2%)	4 (15.4%)	69 (18.9%)	
**Use of NMBA**	243 (71.7%)	18 (69.2%)	261 (71.5%)	0.790
**Type of NMBA**				0.782
Succinylcholine	154 (63.4%)	9 (50.0%)	163 (62.5%)	
Rocuronium	85 (35.0%)	6 (33.3%)	91 (34.9%)	
Missing	4	3	7	
**Use of catecholamines**	130 (38.3%)	6 (23.1%)	136 (37.3%)	0.121
**Environment**				0.137
Easy Access (urban)	273 (80.5%)	24 (92.3%)	297 (81.4%)	
Difficult Access (remote)	66 (19.5%)	2 (7.7%)	68 (18.6%)	
**Night Mission**	33 (9.7%)	4 (15.4%)	37 (10.1%)	0.358

Data was complete if not explicitly stated. Easy Airway Successful first attempt to establish invasive ventilatory support. Difficult Airway Unsuccessful first attempt to establish invasive ventilatory support. *SD* Standard deviation. *CPR* Cardiopulmonary resuscitation. *NACA* National Advisory Committee of Aeronautics. *NMBA* neuromuscular blocking agents

**Fig. 2:**
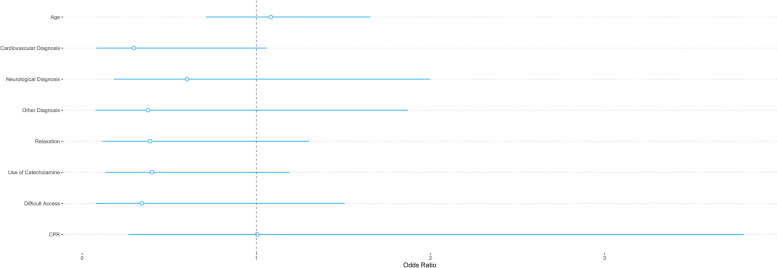
Regression Plot. Illustration of the multivariate logistic regression model summarized in Table 2. Regression coefficients are exponentiated and scaled. The horizontal lines around the dots indicate the 95% confidence interval of the odds ratio. The OR indicates the attributable risk of having the condition. Difficult access = remote location / alpine terrain

The most common condition requiring any form of airway management was trauma (42.7%), followed by cardiovascular emergencies (31.5%) and neurological emergencies (15.9%). Severe traumatic brain injury was the most common indication for out-of-hospital airway management (*n* = 130; 36%). Cardiac arrest requiring CPR was reported in 102 patients (27.9%). The median NACA score was 5 (IQR 5 to 6). There was no significant difference in all other baseline characteristics in both the univariate and the multivariate analyses (Tables 1 and 2, Fig. 2). Mortality on-site and during the HEMS missions was 18.9%, and not significantly different between the two patient groups.

**Table 2:** Logistic regression analysis

Variable	OR	95% CI of OR	p-value
Age in years	1.01	0.99 to 1.03	0.604
Male sex	0.89	0.36 to 2.35	0.797
Diagnosis			
*Trauma*	Ref	Ref	
*Cardiovascular*	0.27	0.06 to 0.99	0.058
*Neurological*	0.62	0.16 to 1.93	0.437
*Other*	0.37	0.05 to 1.53	0.226
CPR	0.93	0.21 to 4.18	0.926
NACA	1.16	0.45 to 3.02	0.755
Use of NMBA	0.43	0.11 to 1.82	0.231
Use of catecholamines	0.38	0.12 to 1.11	0.093
Night mission	1.73	0.45 to 5.42	0.375
Difficult access	0.37	0.06 to 1.32	0.188

Complete case analysis on 365 patients. *OR* Odds Ratio. *CI* Confidence Interval. *CPR* Cardiopulmonary resuscitation. *NACA* National Advisory Committee of Aeronautics. *NMBA* neuromuscular blocking agents

The use of neuromuscular blocking agents to facilitate tracheal intubation was reported in 261 (71.5%) patients. Succinylcholine was used in 163 patients (62.5%) and rocuronium in 91 patients (34.9%). Information about the type of NMBA was missing in seven patients. NMBA was used significantly less often in patients undergoing CPR (29 of 102 patients, 28.4%) compared to patients without CPR (232 of 263, 88.2%), *p* < 0.001.

### Crew experience in HEMS airway management

**Fig. 3:**
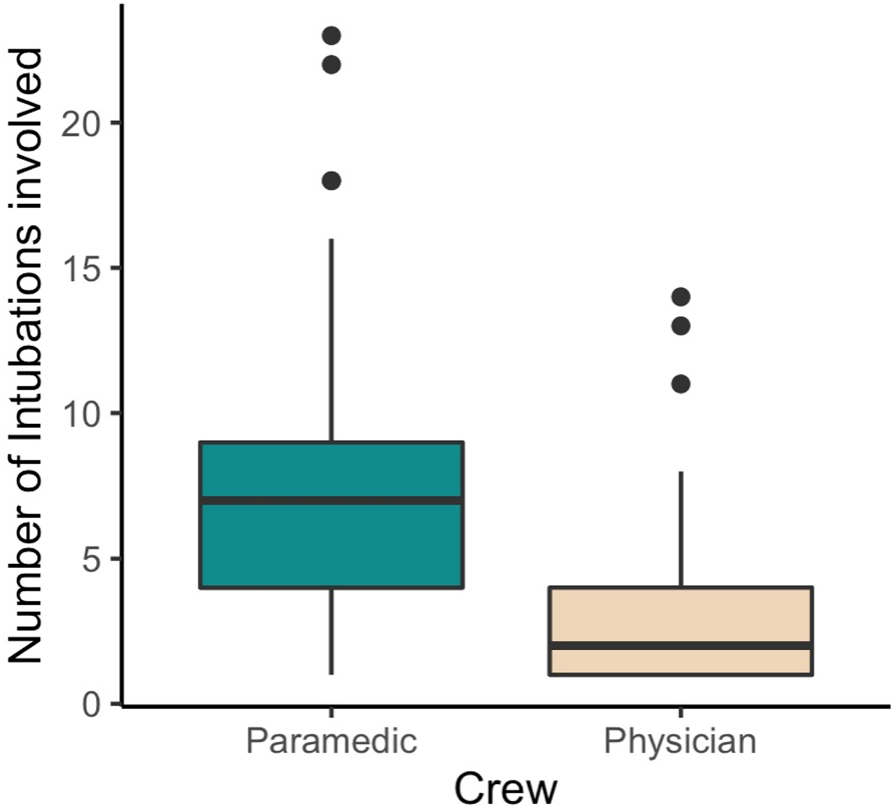
In the observed time period, a total of 129 different Rega physicians treated the patients who needed advanced airway support. Paramedics (*n* = 47) were significantly more involved than physicians in pre-hospital airway manoeuvres: median 7 (IQR 4 to 9) versus 2 (IQR 1 to 4), *p* < 0.001

In the observed period, the 129 different Rega physicians performed a median of 2 (IQR 1 to 4) airway manoeuvres, whereas the 47 Rega paramedics performed a median of 7 (IQR 4 to 9) (see Fig. 3). This difference in crew exposure to airway management in the HEMS setting was significant (*p* < 0.001).

## Discussion

In this cross-sectional study of advanced airway management situations encountered by Rega HEMS over a one-year period, we found a high FPS rate of ETI. Most airway problems could be solved on a second attempt, and only 1.4% (5/365) of patients in the entire cohort of attempted ETIs were unsuccessfully treated or ventilated and oxygenated using another strategy.

### Difficult airway management in HEMS rescues

In this study with an incidence of 7.1%, difficulties in airway management occurred less often than in comparable studies. As reported in a meta-analysis by Lossius et al. [3], the overall out-of-hospital intubation success rate seems to be around 90%, regardless of the number of intubation attempts. In a large prospective study on advanced airway management in HEMS staffed by physicians from different specialties, a first-attempt failure rate of 14.5% was recorded, whereas the overall failure rate was only 1.2% [5]. Although advanced airway management skills play a key role in prehospital medicine, the overall impact of HEMS staffed by physicians is difficult to demonstrate due to a lack of solid evidence [13, 14].

### Patients’ diagnoses

The patients most in need of advanced airway management in our study were trauma patients (42.7%) and patients with cardiovascular emergencies (31.5%). Injuries due to trauma seem to be the predominant reason for providing advanced airway management in many HEMS services around the globe [15, 16]. Early definitive airway protection as well as normoventilation plays a key role in the recent literature on traumatic brain injury (TBI) [16, 17]. This is in accordance with our data showing that TBI was the predominant indication for RSI among all indications.

Some studies have demonstrated lower fractions of trauma patients, such as the study by Gellerfors et al., with data from HEMS services in the Nordic countries. In that study, trauma patients accounted for only 19% of patients receiving ETI, whereas the proportion of patients with cardiovascular emergencies (53%) was higher [4].

Trauma was not associated more often with difficulties in airway management. In contrast to a study by Sunde et al., cardiac arrest patients showed no higher first-attempt failure rates than non-cardiac arrest patients in our study [5]. We do not have an explanation for this, but it may have to do with differences in the timing of ETI during cardiopulmonary resuscitation (CPR) or the general organisation of CPR between services.

Unlike many other airway studies, we included paediatric airway management in our analysis, with no difficulties reported in the nine intubations performed in the age group ≤ 2 years. This is interesting, since this age group is known to be particularly vulnerable to adverse events [13, 23].

### Environmental factors & patient location

It can be assumed that airway manoeuvres might be more challenging in patients treated outdoors at an accident site, where hazardous weather conditions (rain, bright sun, difficult terrain, noise) may be encountered. Importantly, this should not lead to a delay in tracheal intubation if the indication is correct [18].

Our data did not allow a detailed analysis of environmental factors. Still, a summarized analysis of remote environments like alpine terrain was not associated with difficulties in airway management in our data. This is in contrast to the latest data from Knapp et al., who found that being located outdoors had a significant negative effect on the FPS, e.g. due to direct sunlight shining on the videolaryngoscope (VL) screen or suboptimal patient positioning during the FPS intubation with a VL [19, 20]. Theiler et al. previously described this phenomenon and the advantages of the Macintosh laryngoscope over VL in a manikin study [20]. Fogging is an additional factor which seemed to impact FPS in the recent paper by Knapp et. al. [19]. These factors, which impaired the success of VL, demonstrate that although VL is increasingly becoming the gold standard, direct laryngoscopy is still a valuable skill and an important option in difficult situations.

### Solutions for difficult airway management

In cases of failed intubation, supraglottic airway devices were the primary solution in our current data highlighting the importance of these tools as back-up devices [21, 22]. Likewise, BVM ventilation was possible in almost all cases, and remains a valuable option for adequately trained personnel if securing the airway by advanced means is not possible. Nine of the cases with first-pass failure were resolved with an airway introduction catheter (“bougie”). It is unknown whether primary use of this device would have further improved the FPS rate, but other studies have reported an increase in success rates from 85.7 to 98.2% after the introduction of a bougie for use in all out-of-hospital intubations [23, 24]. The use of VL was documented in only 26 cases, 24 of which were managed with a C-MAC STORZ® device, and the other two with an AIRTRAQ. Still, as a consequence of the findings in this study, Rega HEMS has introduced the regular use of VL as the primary intubation aid, in order to increase FPS rates [19, 25].

### Training

As our data illustrates, advanced airway procedures are performed on only a small fraction of patients encountered by HEMS. With one in ten patients receiving advanced airway management and 129 physicians in the service, it is not surprising that each physician encounters a median of only two intubation cases per year. Other studies from similar HEMS systems have demonstrated similarly low numbers for airway management and other advanced skills [26]. To perform complex and time-critical (difficult) airway interventions, the necessary procedural and manual skills have to be trained and perfectly mastered in in-hospital settings (Operation room, intensive care unit, emergency department, etc.) as well as under emergency conditions before a physician can be signed off for HEMS rescue missions [27–31]. Additional training in direct laryngoscopy for Plan B situations, such as in case of a difficult airway, or fogging, blood, and reflections on the video laryngoscope’s screen could be crucial. The impact of level of training and successful airway management has not been explored in this study.

### Limitations

There are several limitations inherent in the retrospective observational study design. By limiting this study to a consecutive cohort of primary missions provided by a single HEMS provider within a reasonably short period of one year, a more homogenous dataset with good quality was achieved. Still, the partially post-hoc documentation of mission details right after the mission is completed brings the risk of recall bias and selection bias. Thus, the use of devices during airway management, the number of intubation attempts performed, and the difficulties encountered may not have been accurately described. Further, the presented regression analysis is challenged by the low number of events, and unaddressed residual confounding must be assumed.

An additional limitation is that prehospital complications (i.e.: hypoxia, hypotension, etc.) were not analysed due to lack of available data, although it is known that FPS and overall success itself are without this information less valuable.

By evaluating all protocols by hand and clarifying all potentially inconsistent documentations with the individual physicians, we tried to address these inherent limitations.

## Conclusions

Despite high overall success rates for endotracheal intubation in our physician-staffed HEMS service, individual physicians get little real-life experience with advanced airway management in the out-of-hospital setting. This highlights the importance of solid competence in anaesthesiology.

## Data Availability

The datasets used and/or analysed during the current study available from the corresponding author on reasonable request.

## Abbreviations

BVM: Bag valve mask
CPR: Cardiopulmonary Resuscitation
GEMS: Ground-Based Emergency Services
ETI: Endotracheal intubation
FPS: First-pass success
Rega: Acronym combining «Rettungsflugwacht» and «Garde aérienne» (translating to "air rescue" in German and French, respectively)
HEMS: Helicopter Emergency Medical Service
NACA: National Advisory Committee for Aeronautics
NMBA: Neuromuscular blocking agents
NIV: Non-invasive ventilatory support
No: Number
TBI: Traumatic brain injury
VL: Videolaryngoscope
